# Supplementary material for: Insights into beta cell regeneration for diabetes via integration of molecular landscapes in human insulinomas
Source: Nat Commun. 2017 Oct 3;8:767. doi: 10.1038/s41467-017-00992-9 (PMC5626682; doi:10.1038/s41467-017-00992-9)
Supplement: Supplementary file 1 — Supplementary Information [file 41467_2017_992_MOESM1_ESM.pdf]

Supplementary Figure 1

Strategy for Integrating Molecular Landscapes in Human Insulinomas

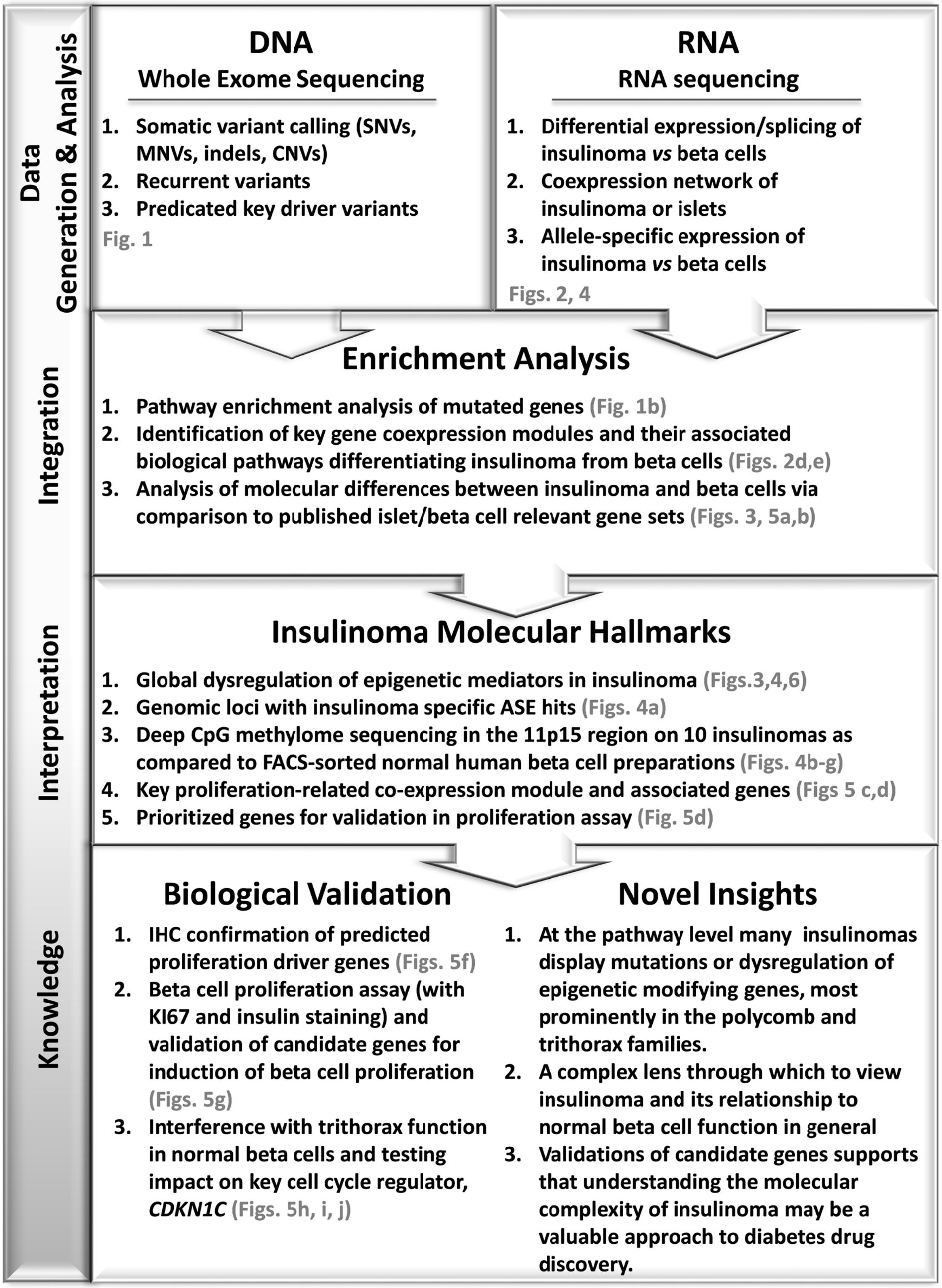

Supplementary Figure 2.

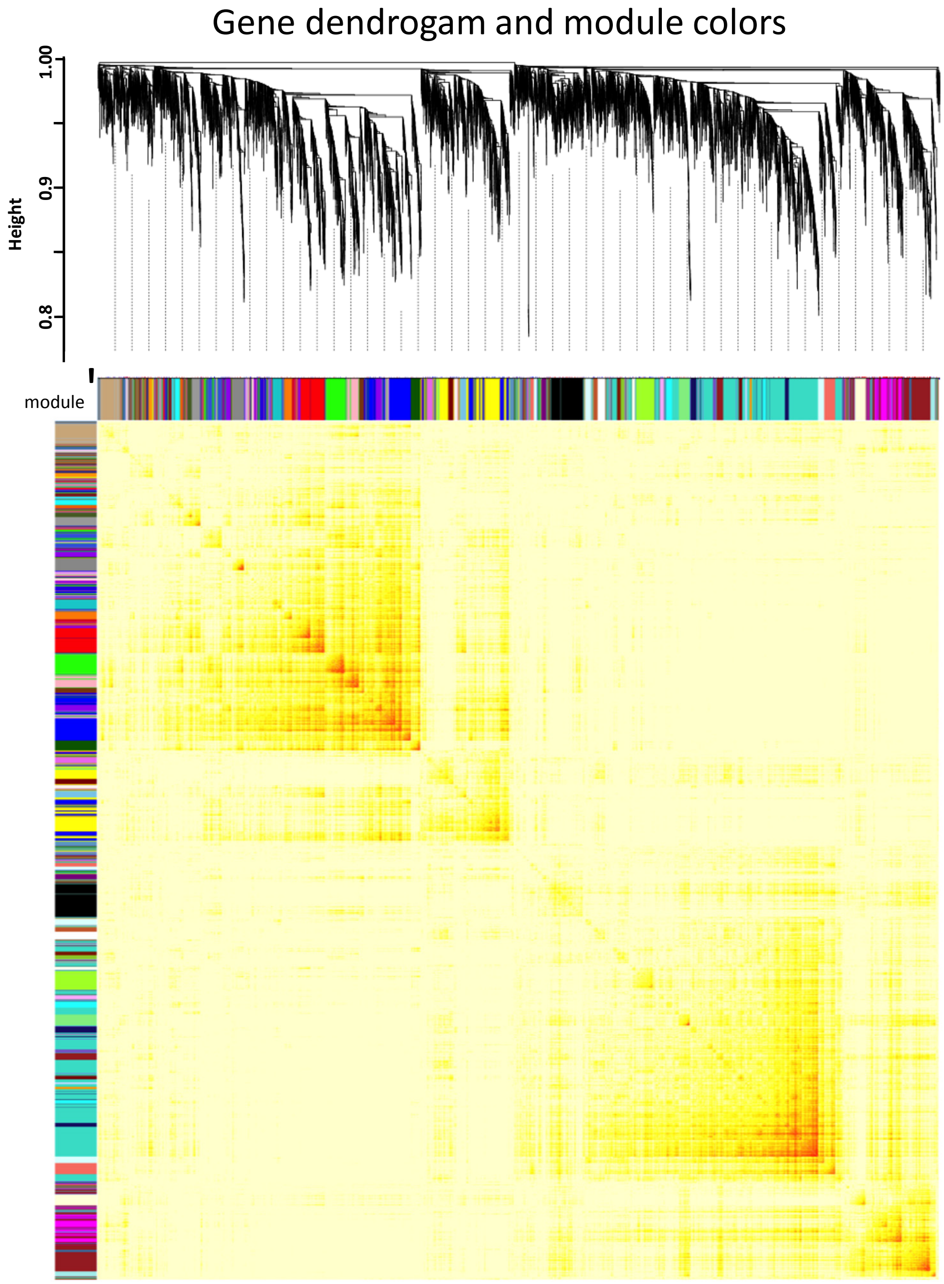

The Complete Insulinoma Co-Expression Network.
Clustering of the Topological Overlap Matrix (TOM) for expressed protein coding genes in insulinomas based on RNAseq data. In the symmetric heatmap, all the genes are represented in rows and columns in the same order. The color intensity reflects the connection strength between two genes, with red representing the strongest signal and white representing the weakest signal. Gene modules were denoted in both x any y axes in different colors.

Supplementary Figure 3

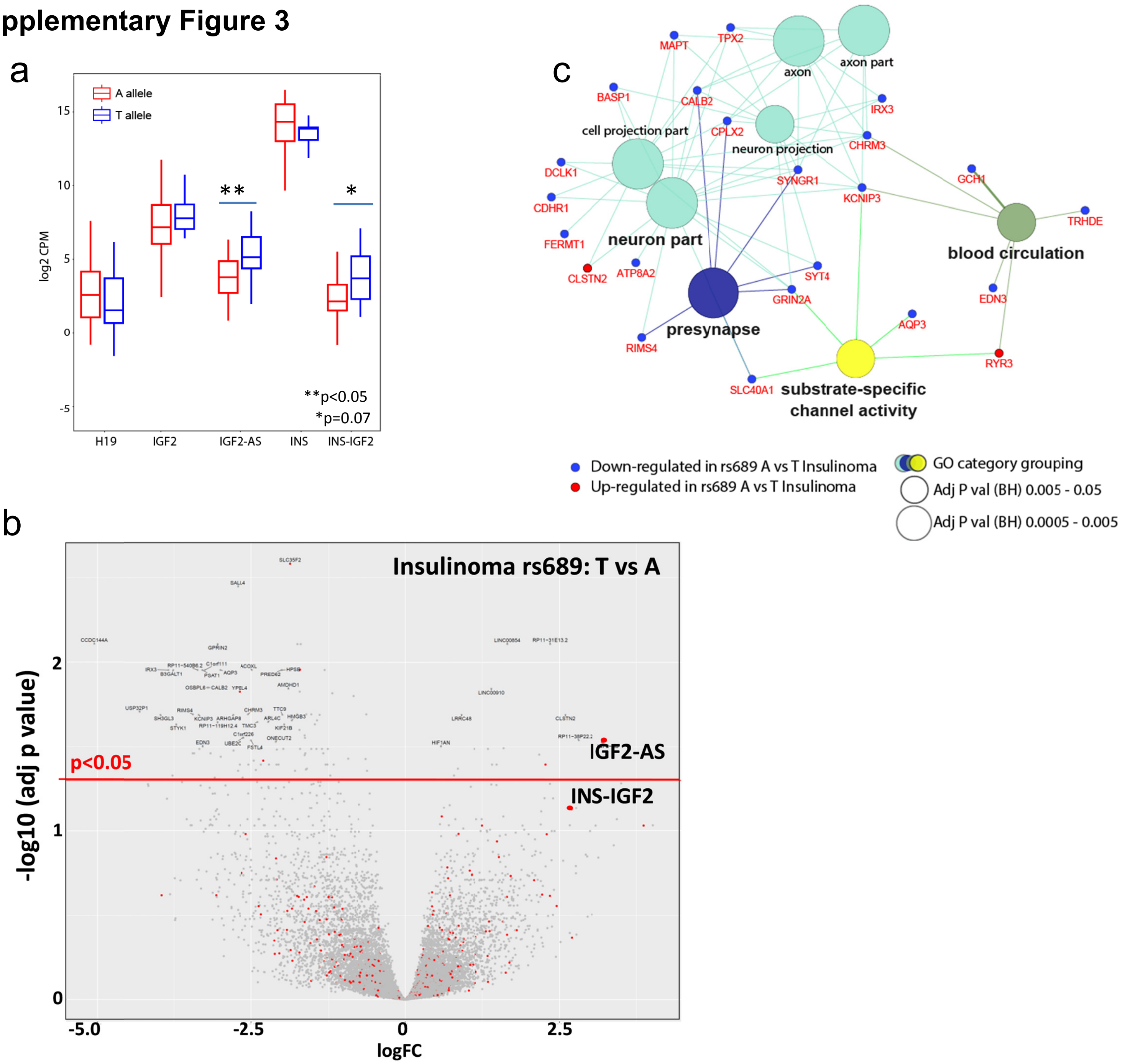

Effects of the rs689 SNP site on chromosome 11 gene expression.

a. Insulinoma samples were separated according to whether they expressed the A (reference allele) (n=17) or the T allele (n=8) at rs689, a SNP within the insulin gene. Differential expression analysis of select genes physically located near insulin revealed higher expression of IGF2-AS and near significant elevation of INS-IGF2 read-through transcript. **b.** Expanded genome-wide differential expression analysis between these two groups revealed 109 differentially expressed genes at adjusted p value >0.05 (Supplementary Data 17). **c.** Pathway enrichment analysis of these 109 differentially expressed genes, performed by ClueGO using GO annotation databases, revealed several significantly enriched pathways. The node and edge plot shows the enriched pathways and the differentially expressed genes found associated with them. Small nodes which are red and blue indicate whether that gene was significantly up- or down- regulated, respectively, in the comparison of T vs A allele-expressing insulinomas. The size of the nodes associated with pathway annotations relate to their respective adjusted p values as indicated while the coloring of the nodes relates to the GO annotation assigned grouping.

Supplementary Figure 4

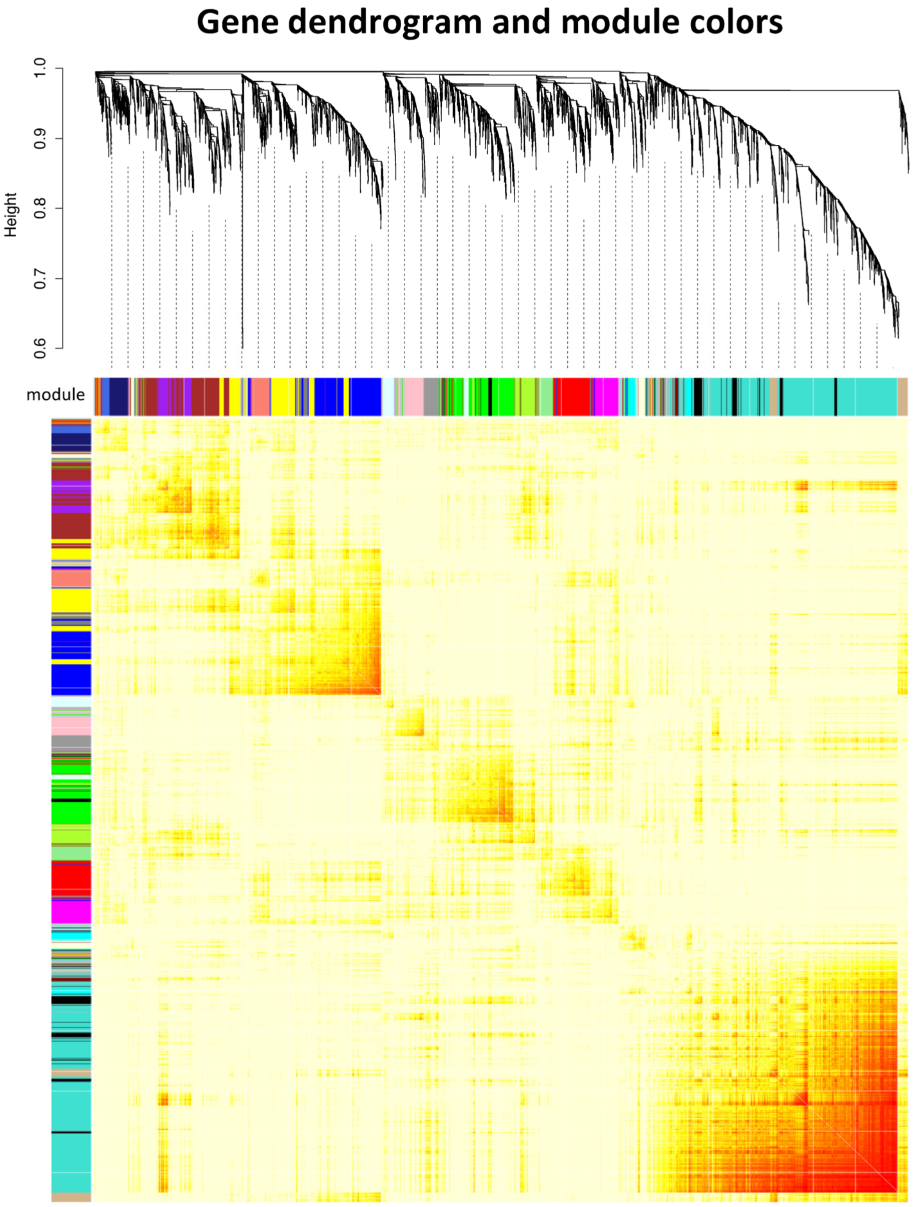

The Complete Human Islet Co-Expression Network.

The same as **Supplementary Figure 2**, but for the human islet RNAseq samples from Fadista et al[30].

Supplementary Figure 5

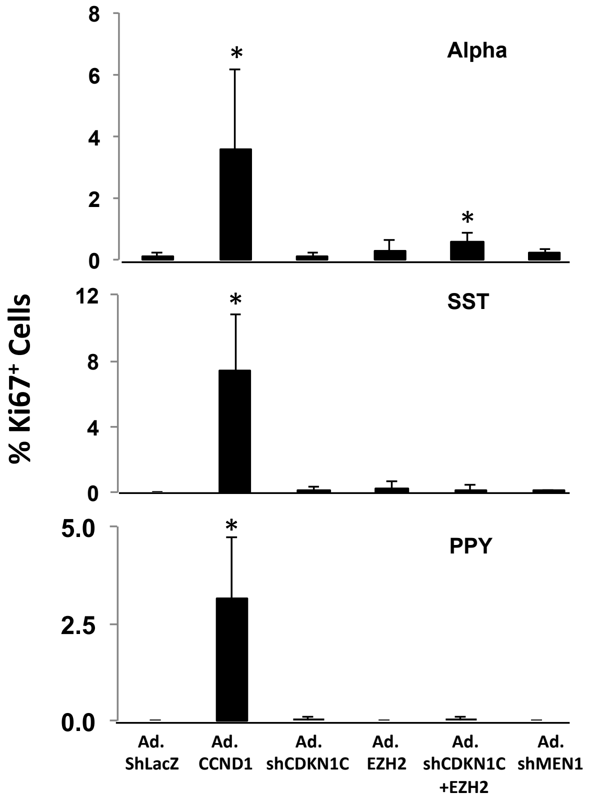

The effect of adenoviral delivery of epigenetic modifiers and cell cycle genes on proliferation in non-beta cells. Ki67 was used as a marker for proliferation, in dispersed human islets, co-immunolabeled with glucagon (alpha cells), somatostatin (delta cells) or PP (PP cells). As expected, *CCND1* induces proliferation in all cells, whereas overexpressing *EZH2*, or silencing *MEN1, CDKN1C* or a combination of *EZH2* overexpression plus *CDKN1C* silencing had no effect. Results are expressed as the percent of the three specific endocrine cell types in which Ki67 labeling was present. A minimum of 1000 cells were counted for each islet cell type. Error bars indicate SEM, and * indicates $p < 0.05$ by Student's unpaired T-test.

Supplementary Figure 6

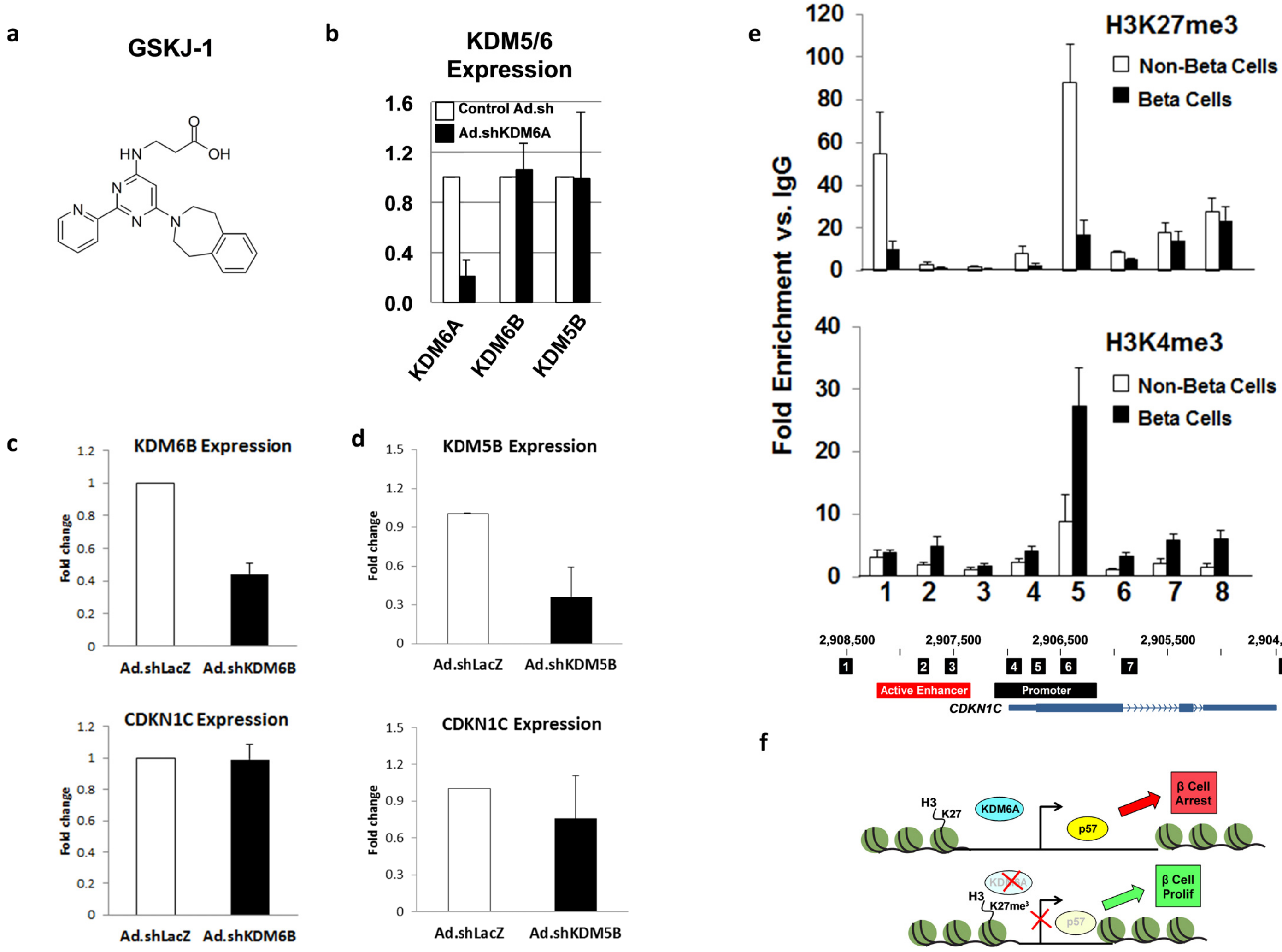

Direct regulation of the cell cycle inhibitor gene, CDKN1C encoding p57^{KIP2} by the trithorax member, KDM6A. **a.** The structure of the pharmacologic *KDM6A* inhibitor, GSKJ-1. **b.** Efficacy of adenoviral silencing *KDM6A* in human islets on expression of *KDM6A*, but not on the related genes *KDM6B* and *KDM5B*, demonstrating the specificity of the Ad.shKDM6A virus on *KDM6A* expression. Error bars indicate SEM. **c.** Lack of effect of adenoviral silencing of *KDM6B* on *CDKN1* expression. Error bars indicate SEM. **d.** Lack of effect of adenoviral silencing of *KDM5B* on *CDKN1C* expression. Error bars indicate SEM. **e.** The upper panel shows ChIP of FACS-sorted beta and non-beta cells for the repressive, polycomb-dependent H3K27me3 mark on the *CDKN1C* region. H3K27me3 is present in upstream and promoter regions of *CDKN1C* in non-beta cells, consistent with the absence of *CDKN1C* expression in non-beta cells. In contrast, this mark is reduced or absent on *CDKN1C* in beta cells, consistent with expression of *CDKN1C* in beta cells. The lower panel shows similar experiments for the open trithorax-dependent chromatin mark, H3K4me3, showing that this region is predicted to be accessible in beta cells, but not non-beta cells, consistent again with *CDKN1C* expression in beta cells, but not other islet cell types. **f.** A cartoon illustrating these changes in beta cells under basal conditions, and (top) and beta cells in which KDM6A has been inhibited with GSKJ-1 or Ad.shKDM6A (bottom), and highlighting the direct interaction of the trithorax member, KDM6A, with the *CDKN1C* locus.

Supplementary Figure 7

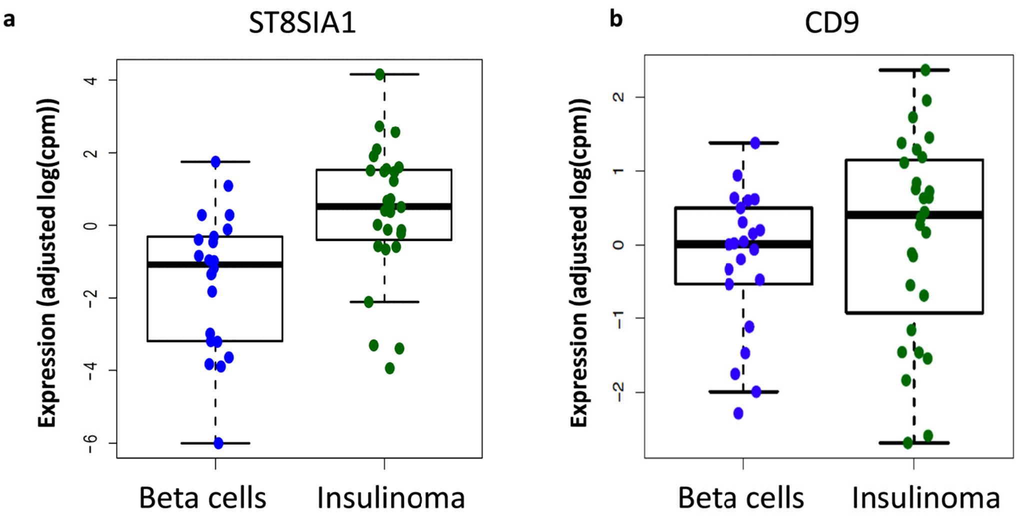

Heterogeneous expression of ST8SIA1 and CD9 in human beta cells and insulinomas. Box and whisker plot of RNAseq or ST8SIA1 and CD9, canonically associated with beta cell heterogeneity[49], expressed as FPKM for each of the two genes among 22 sets of FACS-sorted beta cells and the 25 insulinomas. The data do not allow for assessment of heterogeneity within beta cells in a single islet or insulinoma, but indicate that both beta cells and insulinomas display marked inter-specimen heterogeneity, likely due at least in part, to heterogeneity of cells within a given sample.

Supplementary Figure 8

a Insulinomas vs beta cells

Gene	log2FC	Avg FPKM	FDR
PDGFRA	3.72233	1.93358	3.46E-08
CXCL12	3.61433	3.1339	1.47E-07
LMO4	1.59807	2.16362	0.0000412
SLC16A1	1.45058	1.17362	0.0008609
IGFBP4	1.48303	5.69898	0.0011412
FCGRT	0.861763	4.57001	0.020955
CD302	0.625648	3.09759	0.0699045
TNS1	-0.727501	7.00011	0.161207
C1QBP	0.280112	5.62119	0.29666
LDHA	0.252584	4.9272	0.64934
OAT	0.0802741	5.84896	0.728528
SMAD3	-0.081958	4.56325	0.891364

b Transduced Human Islets

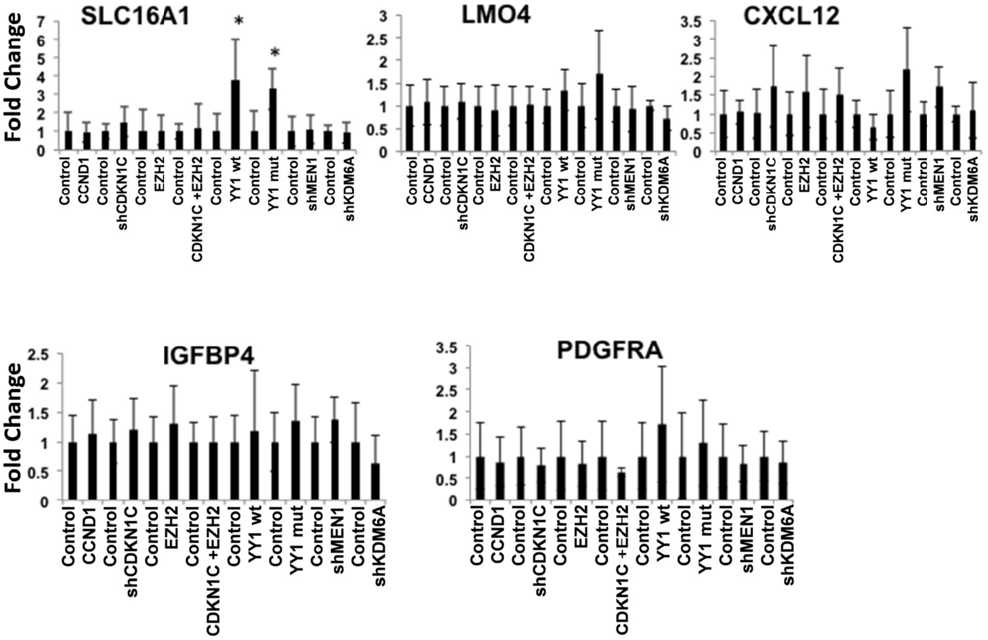

Differential expression of "disallowed genes" in insulinomas (left) and effect of cell cycle and epigenetic manipulation on these genes in normal islets (right). a. Differential expression of candidate disallowed genes in insulinomas vs. beta cells, extracted from **Supplementary Data 8**. The yellow-shaded are statistically significantly increased in insulinomas as compared to FACS-sorted beta cells, and may in theory contribute to the abnormal secretion of insulin in insulinomas. **b.** Effects of adenoviral treatment of human islets with a control adenovirus, or adenoviruses expressing *CCND1, EZH2, YY1,* the Thr372Arg mutant of *YY1*, or encoding shRNAs for *CDKN1C, KDM6A*, or *MEN1* as shown. Among these, only mutant and wild-type *YY1* altered expression of only one "disallowed gene", *SLC16A*, the pyruvate-lactate transporter. Error bars indicate SEM, and * indicates $p < 0.05$ by Student's unpaired T-test.

Supplementary Figure 9

a Insulinomas vs beta cells

Gene	log2FC	Avg FPKM	FDR
SLC2A1	-1.46509	4.3283	9.42E-09
HK3	3.15643	-0.455474	3.95E-08
SLC2A4	3.07589	-0.942312	2.01E-07
SLC2A2	-4.78802	2.62279	5.77E-07
HK1	1.54803	3.16401	0.0016013
SLC2A3	1.22031	4.38292	0.0118842
GCK	-0.723758	6.01771	0.147475
HK2	0.880302	0.594225	0.22354
SLC2A6	-0.1446	4.33046	0.705597
SLC2A5	N/A	<1	N/A

b Transduced Human Islets

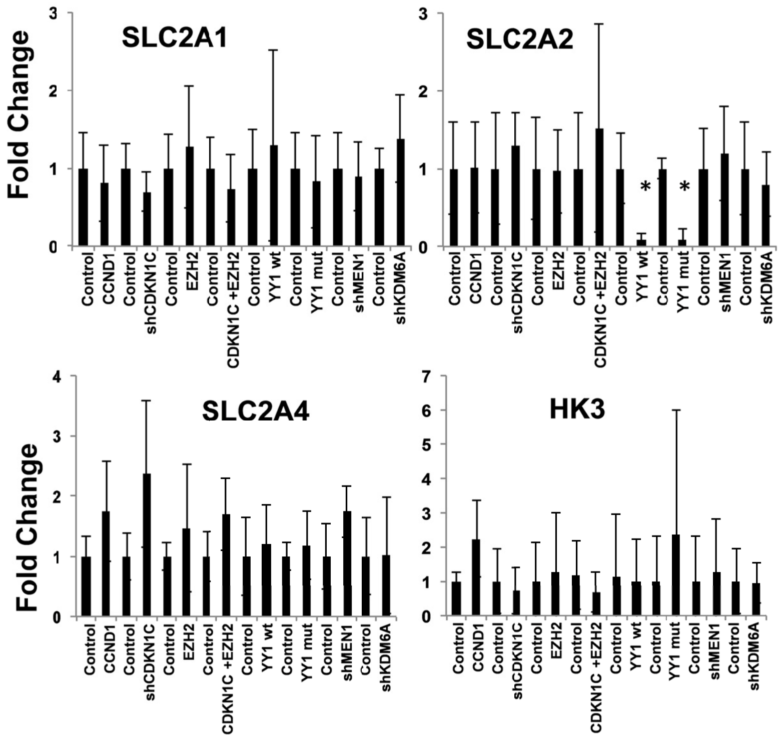

Differential expression of glucose transporters and hexokinases in insulinomas (left) and effect of epigenetic manipulation and effect of cell cycle and epigenetic manipulation on these genes in normal islets (right). a. Differential expression of glucose transporter and hexokinase genes in insulinomas vs. beta cells, extracted from **Supplementary Data 8**. The yellow-shaded are statistically significantly different in insulinomas as compared to FACS-sorted beta cells, and may in theory contribute to the abnormal secretion of insulin in insulinomas. **b.** Effects of adenoviral treatment of human islets with a control adenovirus, or adenoviruses expressing *CCND1, EZH2, YY1,* the Thr372Arg mutant of *YY1*, or encoding shRNAs for *CDKN1C, KDM6A*, or *MEN1*, as shown. Among these, only mutant and wild-type *YY1* altered expression of only one of the genes in this group, *SLC2A2*, encoding the Glut2 transporter. Error bars indicate SEM, and * indicates $p < 0.05$ by Student's unpaired T-test.

Supplementary Figure 10

a Insulinomas vs beta cells

Gene	log2FC	Avg FPKM	FDR
**PDX1**	-1.15702	6.32986	0.0138055
**NKX6-1**	-1.11	6.58	0.0708053
**NEUROD1**	-0.897058	6.53137	0.104482
**ISL1**	-0.545179	6.36173	0.119793
**NKX2-2**	-0.68	5.28	0.308324
**MAF**	0.0552567	4.60599	0.89735
**MAFB**	-0.0838149	7.77628	0.901517
**MAFA**	-0.0906068	6.84971	0.9272
**NEUROG3**	0	<1	N/A
**ARX**	N/A	0-1	N/A

b Transduced Human Islets

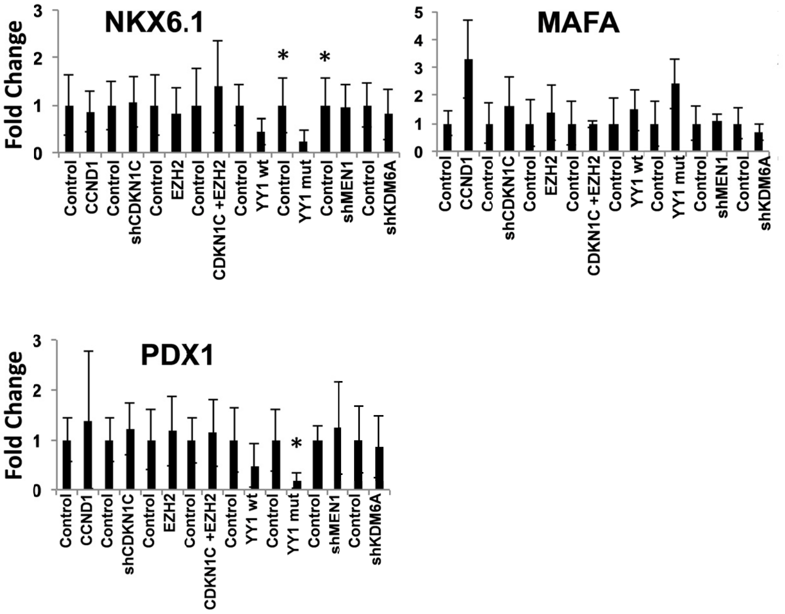

Differential expression of key beta cell transcription factors in insulinomas (left) and effect of epigenetic manipulation and effect of cell cycle and epigenetic manipulation on these genes in normal islets (right). a. Differential expression of transcription factor genes in insulinomas vs. beta cells, extracted from **Supplementary Data 8**. None are strongly different in insulinomas as compared to FACS-sorted beta cells. The minor decreases may reflect contamination of insulinomas with non-beta cell types such as endothelial cells and fibroblasts. **b.** Effects of adenoviral treatment of human islets with a control adenovirus, or adenoviruses expressing *CCND1, EZH2, YY1,* the Thr372Arg mutant of *YY1*, or encoding shRNAs for *CDKN1C, KDM6A*, or *MEN1*, as shown. Among these, only mutant and wild-type *YY1* altered expression of only two of the genes in this group, *NKX6.1* and *PDX1*. Error bars indicate SEM, and * indicates p <0.05 by Student's unpaired T-test.

Supplementary Table 1.

Insulinoma and Patient Characteristics.

Insulinoma #	Age/ Gender	Mt. Sinai ID #	Histology	Diameter	Benign vs. Malig*	Insulinoma DNA Seq	WBC DNA Seq	RNAseq (RIN)	Lowest Blood Glucose/Insulin/ C-Peptide/ProInsulin	Ki67	Hypoglycemia Resolved Postop	FH MEN1
1	53F	4418	Insulinoma	1.4 cm	B	WES	WES	good	31/10.8/?/?	<1%	Yes	No
2	48M	4950	Insulinoma	3.6 cm	B	WES	WES	8.3	34,53/11.5/2.7/?	3-5%	Yes	No
3	73F	4973	Insulinoma	1.9 cm	B	WES	WES	6.7	43/5,13/2.1,2.6/?	<1%	Yes	No
4	82M	4974	Insulinoma	2.2 cm	B	WES	WES	4.7	38/11.9,17.5/7.2,5.4/?	<1%	Yes	No
5	43M	5318	Insulinoma	1.5 cm	B	WES	WES	5.8	47/23/6.63/199.5	2-3%	Yes	No
6	18M	5320	Insulinoma	3.0 cm	B	WES	WES	5.6	33/48/6.55/250.4	7%	Yes	No
7	83M	5322	Insulinoma	1.0 cm	B	WES	WES	N/A	40/3.7/2.47/38	N/A	Yes	No
8	58M	5326	Insulinoma	1.5 cm	B	WES	WES	N/A	<50/35/2867/320	3%	Yes	No
9	59M	5329	Insulinoma	1.0 cm	B	WES	WES	7.4	50/7/7.33/?	<1%	Yes	No
10	57F	5331	Insulinoma	2.2 cm	B	WES	WES	6.9	42/13/2.29/41.1	N/A	Yes	No
11	46M	5333	Insulinoma	2.4 cm	B	WES	WES	7.9	38/<2/1.36/14.2	<2%	Yes	No
12	83M	5404	Insulinoma	1.8 cm	B	WES	WES	N/A	35/37/500/0.48	N/A	N/A	N/A
13	59M	5405	Insulinoma	1.5 cm	B	WES	WES	N/A	N/A	N/A	Yes	N/A
14	45M	5406	Insulinoma	2.7 cm	B	WES	WES	N/A	N/A	0.2 mito/hpf	N/A	N/A
15	65F	5407	Insulinoma	N/A	B	WES	WES	N/A	N/A	N/A	N/A	N/A
16	63F	5408	Insulinoma	N/A	B	WES	WES	N/A	N/A	N/A	N/A	N/A
17	33F	5863	Insulinoma	2.5 cm	M	WES	WES	8.1	36/?/2.2/?	15%	No	No
18	35M	5967	Insulinoma	1.6 cm	B	WES	WES	7.2	49/13.8/11.9/38	2%	Yes	Yes
19	61M	6066	Insulinoma	2.8 cm	B	WES	WES	8.4	42/188/18.7/NA	3-20%	Yes	No
20	35M	6107	Insulinoma	1.5 cm	B	WES	WES	7.7	23/57.7/19.9/38	2%	Yes	No
21	N/A	5564	Insulinoma	N/A	B	no pair	N/A	7.8	no info per IRB	N/A	N/A	N/A
22	N/A	5565	Insulinoma	N/A	B	no pair	N/A	8.6	no info per IRB	N/A	N/A	N/A
23	52F	5594	Insulinoma	N/A	B	no pair	N/A	7.5	39/7.7/2.1/53	N/A	Yes	No
24	48F	5595	Insulinoma	N/A	M	no pair	N/A	7.5	15/85/10.8/1300	N/A	Yes	No
25	29F	5596	Insulinoma	N/A	B	no pair	N/A	5.2	24/19.9/4.5/91	N/A	No	No
26	48F	5597	Insulinoma	N/A	B	no pair	N/A	7.3	36/12.5/6.2/420	N/A	Yes	No
27	32M	5599	Insulinoma	N/A	B	no pair	N/A	7.0	32/92.3/5.6/130	N/A	Yes	No
28	76F	5600	Insulinoma	N/A	B	no pair	N/A	5.9	49/16.8/4.9/210	N/A	Yes	No
29	30M	5601	Insulinoma	N/A	B	no pair	N/A	7.9	42/21.9/5.5/45	N/A	Yes	No
30	30M	5602	Insulinoma	N/A	B	no pair	N/A	8.0	42/21.9/5.5/46	N/A	Yes	No
31	60M	5603	Insulinoma	N/A	B	no pair	N/A	7.0	43/26.2/3.5/480	N/A	Yes	No
32	58F	6957	Insulinoma	1.1 cm	B	no pair	N/A	7.6	46/5/3.4/86.2	<2%	Yes	No
33	60F	8003	Insulinoma	0.9 cm	B	WES	WES	N/A	33/6.5/1.7/4.2	3%	Yes	No
34	71M	9398	Insulinoma	1.0 cm	B	WES	WES	N/A	39/?/?/239	1%	Yes	No
35	65F	BGI.INS15	Insulinoma	N/A	B	WES	WES	N/A	N/A	N/A	N/A	N/A
36	48F	BGI.INS20	Insulinoma	N/A	B	WES	WES	N/A	N/A	N/A	N/A	N/A
37	53M	BGI.INS38	Insulinoma	N/A	B	WES	WES	N/A	N/A	N/A	N/A	N/A
38	53F	BGI.INS40	Insulinoma	N/A	B	WES	WES	N/A	N/A	N/A	N/A	N/A

Notes: **NA** = Not available;
Normal blood glucose = 70-110 mg/dl;
Benign vs Malignant diagnosis is based on pathologist report, although in several cases the Ki67 labeling index exceeds 3%;
No Pair indicates that no WBC DNA was available, so WES was not performed.

Supplementary Table 2.

Predicted Insulinoma Key Driver Variants.

AASS	FLNC	MCC	PVRL1
ADAMTS9	FUT8	MEN1	PZP
ADNP2	GAK	MICAL1	RGS4
AOX1	GLB1	MLH3	RNF111
ARHGAP35	GPS1	MLLT4	SLC27A4
ASB1	GRN	MYOCD	SLK
ATR	H3F3A	NCAM2	SMARCC1
BMP5	HSPA5	NDNL2	SMURF1
BRINP3	IL7R	NEK5	SNX18
CAD	IMPDH1	NLRC3	STAG2
CCDC146	IRAK2	NLRP5	STARD13
CDX2	ITPKB	OTOF	SUFU
CNTNAP4	KATNAL1	P2RY4	TBC1D9B
COL4A5	KATNB1	PAX6	TG
CPNE3	KDM5B	PCDHA9	TMCO1
CREBBP	KDM6A	PER2	TMTC1
CYP4F12	KLHL1	PITPNM2	TPM1
DRD2	KLHL24	PITPNM3	VCP
DSCAML1	KMT2C	PIWIL2	VIM
DUOX2	LIPE	PLCE1	VPS13C
DUSP1	LMO2	PLXNA4	WDR48
ERBB4	MAP3K15	PNPLA3	YY1
ESF1	MAST4	PSIP1	ZBTB44
