# Supplementary material for: Insights into beta cell regeneration for diabetes via integration of molecular landscapes in human insulinomas
Source: Nat Commun. 2017 Oct 3;8:767. doi: 10.1038/s41467-017-00992-9 (PMC5626682; doi:10.1038/s41467-017-00992-9)
Supplement: Supplementary file 3 — Description of Additional Supplementary Files [file 41467_2017_992_MOESM3_ESM.pdf]

Description of Additional Supplementary Files

File Name: Supplementary Data 1
Description: Insulinoma Tumor Sample Whole Exome Sequencing Depth.

File Name: Supplementary Data 2
Description: Protein-Altering SNVs of the Insulinomas.

File Name: Supplementary Data 3
Description: Protein-Altering non-SNVs of the Insulinomas.

File Name: Supplementary Data 4
Description: Pathway Enrichment Analysis of Predicted Insulinoma Key Driver Variants, Based on GO Terms

File Name: Supplementary Data 5
Description: Significant Amplification Peaks by GISTIC2.0 Analysis and Genes Contained within Each Peak

File Name: Supplementary Data 6
Description: Significant Deletion Peaks by GISTIC2.0 Analysis and Genes Contained within Each Peak

File Name: Supplementary Data 7
Description: RNA-seq Sample Sequencing Information

File Name: Supplementary Data 8
Description: Differential Expression Analysis of 13821 genes Expressed in Both Insulinomas and Beta cells, with Beta Cells as the Control.

File Name: Supplementary Data 9
Description: Differential Splicing Analysis (T Test), Insulinoma vs. Beta Cells at FDR 10%

File Name: Supplementary Data 10
Description: Top 100 Genes with Most Significant Differentially Spliced Exons Based on F and Simes Tests

File Name: Supplementary Data 11
Description: Insulinoma Co-Expression Network Table

File Name: Supplementary Data 12
Description: Projection of Protein-Coding DEGs in Isulinomas vs Beta Cells onto the Insulinoma Co-Expression Network.

File Name: Supplementary Data 13
Description: Top 20 GO and KEGG Enriched Terms (based on FDR) for Each Insulinoma WGCNA Module

File Name: Supplementary Data 14
Description: One-Sided Fisher Exact Test for Enrichment of Up-Regulated DEGs in Insulinomas Compared to Beta Cells in Histone Mark Signatures

File Name: Supplementary Data 15
Description: Insulinoma Co-Expression Network Modules Enriched with Histone Marks

File Name: Supplementary Data 16
Description: Allele-Specific Expression (in Both Insulinomas and Beta cells) Result.

File Name: Supplementary Data 17
Description: Differential Expression Analysis of Genes Expressed between rs689 T vs A allele-expressing Insulinomas, with rs689 A allele as the Control

File Name: Supplementary Data 18
Description: Genes with Bisque4 MMP ≤ 0.01
